# Impact of a High-Intensity Training on Ventricular Function in Rats After Acute Myocardial Infarction

**DOI:** 10.5935/abc.20180036

**Published:** 2018-04

**Authors:** Simone de Campos Neitzke Winter, Rafael Michel de Macedo, Júlio Cesar Francisco, Paula Costa Santos, Ana Paula Sarraff Lopes, Leanderson Franco de Meira, Katherine A. Teixeira de Carvalho, José Rocha Faria Neto, Ana Carolina Brandt de Macedo, Luiz César Guarita-Souza

**Affiliations:** 1 Centro de Ciências Biológicas e da Saúde da Pontifícia Universidade Católica do Paraná (PUCPR), Curitiba, PR - Brazil; 2 Instituto Pelé Pequeno Príncipe, Curitiba, PR - Brazil; 3 Universidade Federal do Paraná (UFPR), Curitiba, PR - Brazil

**Keywords:** Myocardial Infarction, Exercise, Ventricular Function, Left, Rats, Anaerobic Threshold

## Abstract

**Background:**

Physical exercise should be part of the treatment of post-acute myocardial infarction (AMI) patients.

**Objective:**

To evaluate the effects of two training prescription models (continuous x interval) and its impact on ventricular function in rats after AMI with normal ventricular function.

**Methods:**

Forty Wistar rats were evaluated by echocardiography 21 days after the AMI. Those with LVEF = 50% (n = 29) were included in the study and randomized to control group (CG n = 10), continuous training group (CTG n = 9) or interval training group (ITG, n = 10). Then, a swimming test with control of lactate production was performed. Based on its result, the lactate threshold (LT) was established to define the training intensities. After six weeks, the animals were reassessed by echocardiography and lactate production. Outcome measures were end-diastolic diameter (EDD), end-systolic diameter (ESD), left ventricular ejection fraction (LVEF, %) lactate at rest, lactate without overload, and lactate with 12g and 13.5g of additional load. Group comparisons of quantitative variables of the study were performed by one-factor analysis of variance (ANOVA). The Newman-Keuls test was used for multiple comparisons of the groups. Within-group comparisons of dependent variables between the two training protocols were performed by Student's t-test. Normality of the variables was tested by the Shapiro-Wilks test. Values of p < 0.05 indicated statistical significance.

**Results:**

EDD, ESD, and LVEF before and after the training period were similar in within-group comparisons. However, EDD was significantly different (p=0.008) in the CG. Significant differences were found for L12g (p=0.002) and L13.5g (p = 0.032) in the ITG, and for L12g (p = 0.014) in the CG. No differences were found in the echocardiographic parameters between the groups. Significant differences were found in lactate without overload (p = 0.016) and L12 (p = 0.031) in the second assessment compared with the first, and between the groups - ITG vs. CG (p = 0.019) and CTG vs. CG (p = 0.035).

**Conclusion:**

Both methods produced a training effect without altering ventricular function.

## Introduction

Cardiovascular diseases (CVDs) are considered the main cause of death in Brazil and in the world in individuals older than 30 years, and acute myocardial infarction (AMI) is responsible for approximately 10% of these deaths.^1^

Treatment after AMI should be pharmacological combined with life habit changes and exercise. Therefore, physical training plays an essential role in AMI treatment.^2^ Current guidelines recommend prescription of physical exercises according to individual's risk stratification, and the most accepted is the combination of moderate-intensity aerobic and resistance exercises.^3^ However, with the progression of prescribed physical training, some authors have decided to prescribe high-intensity training for post-AMI patients.^4^ Experimental studies involving high-intensity training have shown controversial results in terms of benefits to this population.^5,6^

Zhang et al.^5^ investigated the effects of high-intensity sprint training on post-AMI cellular adaptations. Myocytes isolated from hearts with chronic myocardial infarction had a 10% increase in length but not in width, which is consistent with hypertrophy. This may minimize ventricular remodeling and prevent the occurrence of dilated cardiomyopathy.

Benito et al.^6^ used an animal model to evaluate whether sustained intensive exercise training would induce structural changes in the heart. The authors reported cardiac fibrosis after long-term intensive exercise training, together with changes in ventricular function and increased arrhythmia inducibility.

Therefore, the aim of this study was to compare the effects of high-intensity training on post-AMI ventricular function with those of moderate-intensity training.

## Methods

We conducted an experimental study according to the norms and ethical principles of the Brazilian College of Animal Experimentation (COBEA), and approval by the ethics committee for animal research of Pontifical Catholic University of Parana.

First, 40 adult, male Wistar rats weighing 250-300 grams were selected by convenience. The animals had water and food *ad libitum*.

The rats were anesthetized with intramuscular ketamine (Ketamin^®^ / Cristalia - 70 mg/kg) and xylazin (Calmiun^®^/ Agener União- 20 mg/kg). Then, the animals were intubated and mechanically ventilated with oxygen at 2.5 mL/min (small-animal volume). The animals were placed in the supine position (with the body slightly inclined to the right to facilitate the access to the area that would be operated), and all four limbs were fixed using adhesive tape. The chest was shaved and disinfected with povidone-iodine. A left posterolateral thoracotomy was performed at the third intercostal space; as the left pleura was open, the pericardium was removed to expose the operation site. The left auricle was isolated, and the left coronary artery, identified between the pulmonary artery and the left atrium, was ligated with a blue, non-absorbable 7.0 monofilament polypropylene suture thread. The infarcted area was identified by its different color. The heart was then repositioned within the chest, the lungs hyperinflated and the thoracic wall sutured using a non-absorbable, 4.0 nylon monofilament.^7^

Two M-mode echocardiographic examinations (MyLab 40, Esaote^®^) were performed with a 7.5-10.0 MHz sector transducer. The parameters analyzed were left ventricular ejection fraction (LVEF[%]), end-diastolic diameter (EDD[mL]) and end-systolic diameter (ESD[mL]).

Rats with a LVEF > 50% in the first echocardiographic exam were included in the study. The sample was composed of 29 animals, which were randomized using piece of folded papers inside a white envelope. The envelopes were drawn by the main author, and the animals allocated to one of the three groups - control group (CG, n = 10), continuous training group (CTG, n = 9), and interval training group (ITG, n = 10).

The ideal training intensity was determined by a swimming test, with incremental load and control of lactate production. The animals were put in a tank filled to a depth of 40 cm of water (deep enough to prevent the animals to sustain their bodies with their tails on the bottom).^7^ Then, the rats underwent swimming exercise with progressive, additional load (proportional to body weight) - 4.0; 4.5; 5.0; 5.5 e 6.0% of body weight for five minutes each.^8^ The main purpose of this test was to determine the lactate threshold (LT), which was used as the cutoff point for the continuous and interval training loads. Then 25 µL of blood samples were collected from the tail of the animal at rest and at each load progression.^9,10^ Lactic acid production was analyzed using a portable lactate analyzer (Accutrend^®^).

Lactate concentration values were organized in an excel spreadsheet, and a line graph obtained for each animal. LT was visually identified and defined as the point where linearity was lost. This process was performed for both training groups in the swimming tests, one day after each echocardiographic examination.

According to the lactate test results, training intensities prescribed to the CTG and the ITG were at the LT and above the LT, respectively. The CG did not undergo physical training.

Training program of CTG and ITG consisted of a 42-day macrocycle, divided into six 7-day microcycles of 30 exercise sessions (five a week, once a day). The overload method defined for both groups was of volume, i.e., a weekly increase in the time of swimming (min). In the first two weeks of training, CTG underwent swimming training for 10 minutes continuously. In the third and fourth week, the rats swam for 15 minutes, and for 20 minutes in the last two weeks. The ITG underwent five 2-minute sessions with 2-minute intervals between them and 1:1 training density in the first two weeks. In the third and fourth weeks, the rats swam seven 2-minute sessions, with the same interval between them. Finally, in the two last weeks, the animals swam ten 2-minute series, with the same interval between them.

Our outcome measures were LVEF, ESD, ESD and the effect of training (lactate curve). Within-group and between group comparisons of these parameters were performed by a blinded investigator.^9^

At the end of the experiment, the animals were euthanized by sodium pentobarbital (i.v. 200-250 mg/Kg).

### Statistical analysis

Continuous variables were expressed as mean ± standard deviation. Comparisons of quantitative variables were performed by one-factor analysis of variance (ANOVA), and the Newman-Keuls test was used for multiple comparisons. Comparisons between the two evaluations within each group were performed by Student's t-test for dependent variables. Normality of the variables was tested by the Shapiro-Wilks test. Statistical significance was set at p < 0.05. Analyses were performed using the Statistica software, version 8.0.

## Results

Pre- and post-training echocardiographic results and results of lactate tests were compared within and between groups.

Tables 1 and 2 describes the results of within and between group comparisons, respectively, of LFEV, and left ventricular EDD and ESD, and Tables 3 and 4 describes the results of within and between group comparisons, respectively, of pre- and post-training results of lactate testing with incremental load.

**Table 1:** Within-group echocardiographic comparison of mean left ventricular ejection fraction, end-diastolic diameter and end-systolic diameter

GROUP	EDD1	EDD2	p	ESD1	ESD2	p	LVEF1	LVEF2	p
CG	0.26	0.13	0.008*	0.17	0.74	0.120	76.10	71.20	0.112
CTG	0.50	0.58	0.741	0.83	0.19	0.422	73.67	71.89	0.579
ITG	0.19	0.88	0.153	0.78	0.01	0.510	70.70	71.50	0.792

CG: control group, CTG: continuous training group; ITG: interval training group; EDD1: end-diastolic diameter at first evaluation; EDD2: end-diastolic diameter at the second evaluation; ESD1: end-systolic diameter at first evaluation; ESD2: end-systolic diameter at second evaluation; LVEF1: left ventricular ejection fraction at first evaluation; LVEF2: left ventricular ejection fraction at second evaluation; p = p-value of LVEF between the two study days. Student's t-test,

* p < 0.05.

**Table 2:** Between-group echocardiographic comparisons of left ventricular ejection fraction, end-diastolic diameter and end-systolic diameter

Variable	Group	Mean ± SD	p
LVEF1 (%)	CG	76.10 ± 6.89	0.368
	CTG	73.67 ± 10.01	
	ITG	70.70 ± 8.15	
LVEF 2 (%)	CG	71.20 ± 6.44	0.981
	CTG	71.89 ± 8.68	
	ITG	71.50 ± 7.53	
EDD 1 (mm)	CG	5.26 ± 0.80	0.103
	CTG	6.50 ± 1.63	
	ITG	6.19 ± 1.30	
EDD2 (mm)	CG	6.20 ± 0.58	0.404
	CTG	6.00 ± 1.15	
	ITG	6.00 ± 1.69	
ESD1 (mm)	CG	3.17 ± 0.70	0.308
	CTG	3.83 ± 0.93	
	ITG	3.78 ± 1.40	
ESD 2 (mm)	CG	3.74 ± 0.75	0.709
	CTG	4.19 ± 1.23	
	ITG	4.01 ± 1.46	

LVEF1: left ventricular ejection fraction at first evaluation; LVEF2: left ventricular ejection fraction at second evaluation; p: p-value of LVEF between the two study days EDD1: end-diastolic diameter at first evaluation; EDD2: end-diastolic diameter at the second evaluation; ESD1: end-systolic diameter at first evaluation; ESD2: end‑systolic diameter at second evaluation; CG: control group, CTG: continuous training group; ITG: interval training group; One-factor ANOVA

**Table 3:** Within-group comparisons of pre- and post-training lactate test parameters

Variables	Group	N	Mean T1 (SD)	Mean T2 (SD)	p
LR	CG	10	3.90 ± 1.07	4.32 ± 0.47	0.240
	CTG	9	3.83 ± 0.96	3.96 ± 0.22	0.720
	ITG	10	4.18 ± 0.81	4.24 ± 0.32	0.830
LO	CG	10	5.92 ± 1.11	5.99 ± 0.74	0.850
	CTG	9	5.90 ± 2.26	5.07 ± 0.88	0.392
	ITG	10	5.96 ± 1.04	5.18 ± 0.47	0.084
L12g	CG	10	6.58 ± 1.16	6.76 ± 1.04	0.735
	CTG	9	7.32 ± 1.83	5.66 ± 1.06	0.062
	ITG	10	8.08 ± 1.56	5.82 ± 0.65	0.002*
L13.5g	CG	10	6.80 ± 1.32	6.52 ± 1.80	0.733
	CTG	9	8.11 ± 2.14	5.67 ± 0.92	0.014*
	ITG	10	8.40 ± 2.28	5.97 ± 0.80	0.032*

CG: control group, CTG: continuous training group; ITG: interval training group; SD: standard deviation; LR: lactate at rest; LO: lactate without overload; L12g: lactate with 12 grams of additional load; L13.5g: lactate with 13.5 grams of additional load. Student's t test.

* p < 0.05.

**Table 4:** Between-group comparison of lactate test parameters

Variable	Group	n	Mean ± SD	p-value
LWO 1	CG	10	5.92 ± 1.11	0.996
	CTG	9	5.90 ± 2.26	
	ITG	10	5.96 ± 1.04	
LWO 2	CG	10	5.99 ± 0.74	0.016*
	CTG	9	5.07 ± 0.88	
	ITG	10	5.18 ± 0.47	
L 12 g 1	CG	10	6.58 ± 1.16	0.110
	CTG	9	7.32 ± 1.83	
	ITG	10	8.08 ± 1.56	
L 12g 2	CG	10	6.76 ± 1.04	0.031*
	CTG	9	5.66 ± 1.06	
	ITG	10	5.82 ± 0.65	
L 13.5g 1	CG	10	6.80 ± 1.32	0.176
	CTG	9	8.11 ± 2.14	
	ITG	10	8.40 ± 2.28	
L 13.5 g 2	CG	10	6.52 ± 1.80	0.341
	CTG	9	5.67 ± 0.92	
	ITG	10	5.97 ± 0.80	

CG: control group; CTG: continuous training group; ITG:interval training group; SD: standard deviation; LR: lactate at rest; LWO: lactate without overload; L12g: lactate with 12 grams of additional load; L13.5g: lactate with 13.5 grams of additional load. 1: first evaluation; 2: final evaluation. One-factor ANOVA

* p < 0.05.

**Graph 1:**
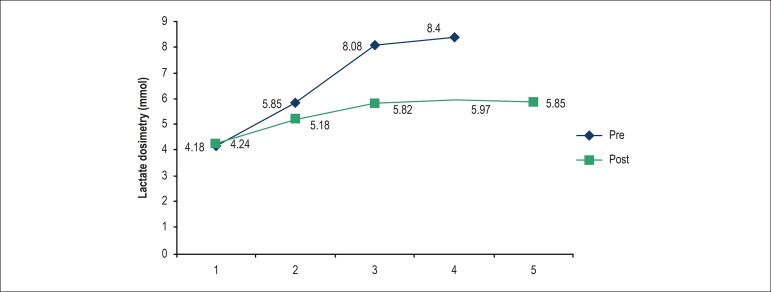
Comparison between the pre and post-training CTG lactate tests.

**Graph 2:**
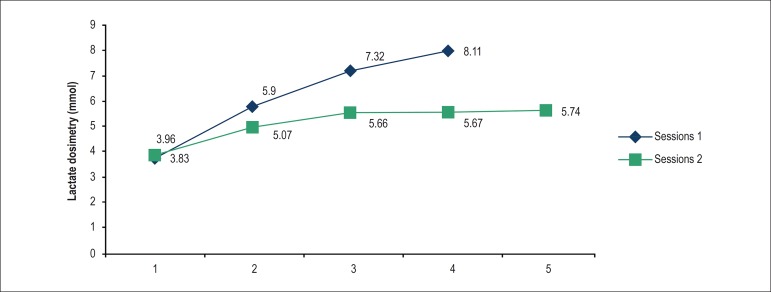
Comparison between pre-versus post-training CTG.

Graphs 1 and 2 show comparative results of pre-training *versus* post-training lactate in ITG and CTG, respectively.

## Discussion

The main findings of the present study were: 1) No differences were found in within-group and between-group comparisons of echocardiographic parameters in CTG and ITG; 2) A worsening of EDD was observed in the CG; 3) Both groups subjected to exercise showed significant differences in lactate production in the pre- *versus* post-training periods: ITG for the loads L12g and L13.5g, and CTG for L12g; 4) No difference was found between initial and final tests in the CG.

The lack of difference in the pre- and post-training period between ITC and CTG indicates that high-intensity training, above LT, may be recommended for this sample. Current national and international guidelines^1,3^ recommend a moderate-intensity, predominantly aerobic training (between the ventilatory thresholds, when evaluated by ergospirometry), i.e., below LT, to post-AMI patients. If these findings were extended to humans, these patients would benefit from this type of training, since it enables higher energy expenditure, better cardiovascular fitness and hence better control of cardiovascular risk factors.^11^ Nevertheless, the same cannot be affirmed for animals with reduced LVEF, and further studies with the same study design are needed to assess the impact of a high-intensity training on ventricular remodeling.

It is worth mentioning that an inadequate volume/intensity load of cardiac training or exercise may be assessed by changes in ventricular wall kinetics,^12^ as evaluated by Neilan et al.,^13^ in a study with nonelite participants in the Boston marathon who were less trained. These findings were not observed in our trained groups, which implies that training density (relationship between volume and intensity) was well distributed. In addition, the training protocol proposed in this study may be applied to post-AMI patients with LVEF ≥ 50%, as long as a training/interval ratio of 1:1 for aerobic training and 1:2 for high-intensity interval training were respected.

The only significant change detected in the echocardiographic analysis after the study was the increase in EDD (p = 0.008) in the CG, which suggests that, after a six-week period of rest, these animals had an unfavorable ventricular remodeling when compared to the other groups.

In an experimental study by Gaudron et al.,^14^ 156 rats were randomized after coronary occlusion into three groups - sedentary, those who started training 4 days after AMI and those who started training 21 days after AMI. The aim of the study was to assess the influence of continuous training (8 weeks' duration), initiated early or delayed, on ventricular function and mortality. The authors demonstrated that 1) neither infarction nor exercise had any effect on the animals' survival; 2) in rats with small infarcts, left ventricular volume and shape, and long-term survival were not altered by chronic exercise initiated early or late after coronary artery ligation; 3) Mortality rose in animals with large infarction as a result of exercise (p < 0.0001) and was 47.6% with early exercise and 26.7% with late exercise (p < 0.05, early *versus* late).

It is of note that findings of left ventricular volume reported by Gaudron et al.^14^ are similar to those described in our study, since no differences between pre- and post-training in cavity diameter were found in the training groups (ITG and CTG). On the other hand, mortality rates were different, since no deaths occurred in the present study. This may be explained by different training volumes between their study by Gaudron et al.,^14^ and ours; in their study, the animals underwent continuous training for 90 minutes, with no progression or periodization program, whereas in our study, the maximum training period was 20 minutes, completed after a periodization program with progressive loads.

The development of a training program in a subjective manner, without load (intensity and/or volume) individualization or progression, and without temporal organization (periodization) should be considered inadequate, since the effects of training may be underestimated by an arbitrary exercise prescription. Therefore, studies in which exercises are prescribed in such arbitrary manner may yield inconsistent results, as exercises may be less effective than expected.

Therefore, aiming to provide the most effective exercise prescription, we elaborated an individualized method of exercise assessment and prescription. First, the animals underwent a LT test with incremental load before training. Based on this LT results and training group allocation (CTG or ITG), the optimal training load of each animal was defined. The model of load progression adopted was the volume progression (every two weeks) according to the pre-established periodization.

At the end of the training program, the LT test was repeated aiming to evaluate the effect of the training. When pre- and post-training results were compared in the ITG and CTG (intragroup comparison), the LT graph moved to the right (Graphs 1 and 2), indicating a positive effect of the training, i.e., the animals can tolerate a higher training load with similar energy consumption. Anaerobic threshold has been used as a measurement of physical fitness to assess the effects of training in patients with CVD and in healthy subjects, acting as a sensitive indicator of aerobic conditioning.^15^ In addition, measurement of LT establishes an effective training intensity in terms of aerobic metabolic dynamics of active muscles. This training effect behavior has a high practical applicability, as improvements in physical fitness may be detected during the training sessions.^16^

However, between-group comparisons did not show any significant differences between ITG and CTG (Table 4), which indicates that both models had a similar effect in this sample. Besides, as expected, no differences were found between the results before and after the training period in the CG, as favorable effects of training cannot be produced during resting condition. Also, Table 1 shows significant differences between the training groups (CTG and ITG) and the CG in two-by-two comparisons.

The fact that the ITG and the CTG showed similar results after the training period may be justified by studies^17,18^ that support that there is no evidence of the superiority of one exercise prescription model over another one in improving aerobic conditioning. However, the study by Vona et al.^11^ concluded that both methods or their combination are efficient and safe to correct endothelial dysfunction in recent AMI. Schjerve et al.^12^ demonstrated that high-intensity interval exercise was more effective in improving endothelial function and in reducing cardiovascular risk than moderate-intensity continuous exercise.

It is worth pointing out that high-intensity training tends to have a better effect on maxVO_2_ and lactate tolerance than on anaerobic threshold (or LT); in contrast, continuous training improves LT, but not necessarily peak VO_2_. Since the aim of the incremental test in this study was to determine the LT and thereby establish the optimal training load, changes in maximum physical capacity were not evaluated, which could be favored by the interval training.

Since interval training has been recently investigated in cardiac rehabilitation programs and periodization of this type of exercise has not been well defined, it is possible that changes in the number of training repetitions and resting periods may produce more positive and favorable results than continuous training.^19^ We believe that the same training prescription used for the animals in the present study may be performed for post-AMI patients in rehabilitation programs.

## Conclusion

This study demonstrated that high-intensity training, above the LT, did not worsen endothelial function, and was safe for post-AMI rats. Both training methods proposed improved cardiorespiratory fitness in the animals.

### Study limiation

One possible limitation of this study was the use of a portable lactate analyzer instead of a micropipette.
